# Giant Enhancement of Magnetostrictive Response in Directionally-Solidified Fe_83_Ga_17_Er*_x_* Compounds

**DOI:** 10.3390/ma11061039

**Published:** 2018-06-19

**Authors:** Radhika Barua, Parisa Taheri, Yajie Chen, Anjela Koblischka-Veneva, Michael R. Koblischka, Liping Jiang, Vincent G. Harris

**Affiliations:** 1 College of Engineering, Northeastern University, Boston, MA 02115, USA; paris.t14@gmail.com (P.T.); y.chen@neu.edu (Y.C.); 2 Institute of Experimental Physics, Saarland University, 66123 Saarbrucken, Germany; a.koblischka@gmail.com (A.K.-V); m.koblischka@mx.uni-saarland.de (M.R.K.); 3 Baotou Research Institute of Rare Earths, Baotou 014010 China; btjlp@126.com

**Keywords:** iron-gallium, magnetostriction, rare-earth doped FeGa

## Abstract

We report, for the first time, correlations between crystal structure, microstructure and magnetofunctional response in directionally solidified [110]-textured Fe_83_Ga_17_Er*_x_* (0 < *x* < 1.2) alloys. The morphology of the doped samples consists of columnar grains, mainly composed of a matrix phase and precipitates of a secondary phase deposited along the grain boundary region. An enhancement of more than ~275% from ~45 to 170 ppm is observed in the saturation magnetostriction value (*λ_s_*) of Fe_83_Ga_17_Er_x_ alloys with the introduction of small amounts of Er. Moreover, it was noted that the low field derivative of magnetostriction with respect to an applied magnetic field *(*$i.e.,\;d\lambda_{s}/dH_{app}$ for *H_app_* up to 1000 Oe) increases by ~230% with Er doping ($d\lambda_{s}/dH_{app,\mathrm{FeGa}}=$ 0.045 ppm/Oe; $d\lambda_{s}/dH_{app,\mathrm{FeGaEr}}=$ 0.15 ppm/Oe). The enhanced magnetostrictive response of the Fe_83_Ga_17_Er*_x_* alloys is ascribed to an amalgamation of microstructural and electronic factors, namely: (i) improved grain orientation and local strain effects due to deposition of Er in the intergranular region; and (ii) strong local magnetocrystalline anisotropy, due to the highly anisotropic localized nature of the *4f* electronic charge distribution of the Er atom. Overall, this work provides guidelines for further improving galfenol-based materials systems for diverse applications in the power and energy sector.

## 1. Introduction

Functional materials systems that demonstrate large magnetostriction play an important role in a wide array of commercial applications, ranging from acoustic sensors and linear actuators to electromechanical energy harvesters and sonar transducers [1,2,3]. One of the most successful magnetostrictive materials hitherto is the rare-earth compound, (Dy_0.7_Tb_0.3_)Fe_2_ (also known as Terfenol D) [4]. These alloys demonstrate a cubic C15 laves crystal structure and exhibit large room temperature magnetic-field-induced strains up to 2000 ppm [4]. It is well known that Terfenol D has several major drawbacks that constrain its use in commercial devices, particularly: (i) high cost and global shortage of the rare-earth elements, Tb and Dy [5]; (ii) low mechanical integrity (high brittleness, low yield stress, low magneto-mechanical coupling) [6]; and (iii) high fields required for magnetic saturation [7]. To this end, a promising alternative to (Dy_0.7_Tb_0.3_)Fe_2_ is the rare-earth-free, inexpensive and corrosion resistant Fe_1−*x*_Ga*_x_* alloy (commercially known as Galfenol), which exhibits moderate magnetostriction (up to approximately 400 ppm) under a very low magnetic field of 100 Oe and a high tensile strength of 500 MPa in the temperature range from −20 to 80 °C [8].

An intriguing characteristic of Fe_1−*x*_Ga*_x_* is that its functional response is closely correlated with its microstructural and crystallographic properties [8]. The binary phase diagram of Fe_1−*x*_Ga*_x_* indicates that the single-phase terminal solid solution possesses a chemically disordered body-centered cubic (bcc) crystal structure that extends to Ga concentrations of 11 at.% at room temperature and to as much as 35 at.% Ga at 1050 °C [9]. In the composition range (~27–28 at.% Ga), the alloy also exists as Fe_3_Ga, and it exhibits a chemically-ordered cubic L1_2_ crystal structure that undergoes polymorphic transformations to the ordered hexagonal D0_19_ and cubic D0_3_ phase upon heating [10,11]. Further, a B2 ordered cubic phase variant is also noted at high temperatures for compositions exceeding 32 at.% Ga [10]. Depending upon the sample synthesis and the processing technique employed, significant amounts of Ga (well in excess of the solubility limit) can be retained in a metastable disordered bcc solid solution at room temperature [8,10,12]. High magnetostriction values ranging from 250 to 400 ppm have been reported in single crystals of bcc alloys of Fe_1−*x*_Ga*_x_*_,_, where *x* ranges from 15 to 25 [13].

Over the last twenty years, attempts have been made to improve the magnetostrictive response of galfenol by addition of a wide variety of elements into its crystal lattice. In almost all cases, ternary additions of *3d* and *4d* transition metals (V, Cr, Mn, Co, Ni, Rh and Mo) decrease the magnetostriction values, relative to that of the parent binary FeGa alloy [14,15,16,17,18]. While tiny amounts of small interstitial atoms (C, B or N) have a slight but favorable effect on the magnetostriction of FeGa (particularly at high atomic compositions of Ga) [18,19]. More recent studies indicate that the magnetostriction of this compound can be significantly increased by adding small amounts of rare-earth elements ranging from La to Lu [20,21,22,23,24,25,26,27,28]. A phenomenological model based on the rare-earth crystal field interaction, proposed by He et al., suggests that the best trace dopants are the light rare earths, Ce and Pr (up to 0.2 at.% doping), which give a transverse magnetostriction of up to 800 ppm [24]. However, to date, very little attention has been given to Er-doped FeGa compounds.

To add to the FeGa literature, here, we present, for the first time, an experimental study that aims at investigating the crystallographic, microstructural, magnetic and magnetostrictive properties of a series of Er-doped polycrystalline alloys of composition, Fe_83_Ga_17_Er*_x_* (0 < *x* < 1.2). Results obtained in this research effort represent an exceptional increase (~275%) in the magnetostriction coefficient of [110]-textured FeGa alloys. The optimal composition for the best magnetostrictive response was found to be Fe_83_Ga_17_Er*_x_* (*x* = 0.6). The origin of the enhanced magnetostrictive effect in this materials system is discussed in the context of the microstructure, as well as the electronic structure of samples. Overall, this work provides pathways for enhancing the functional response of FeGa alloys.

## 2. Materials and Methods

Polycrystalline samples of nominal composition Fe_83_Ga_17_Er*_x_* (0 < *x* < 1.2) were synthesized from constituent elements of 99.9% purity, using vacuum arc-melting and directional-solidification techniques. The bulk ingots were subsequently placed in an Ar atmosphere and annealed at 900 °C for two hours to obtain the desired phase and microstructure. The arc-melted charges were then sliced into cuboid-shaped slabs (dimensions: 0.01 m × 0.01 m × 0.001 m) using a low-speed diamond saw for characterization of structural, magnetic and magnetostrictive attributes.

Microstructural analysis was carried out on mechanically polished sample slices using an Electron Backscatter Diffractometer, consisting of a JEOL 700F SEM microscope (JEOL, Welwyn Garden City, UK) equipped with a TSL OIM analysis unit (AMETEK, Leicester, UK) and a laboratory CuK_α_ X-ray diffractometer (Rigaku Ultima III, Wilmington, MA, USA). Bragg peaks obtained from the X-ray diffraction pattern were least-squares fit to a Pseudo-Voigt function to estimate lattice parameters of the Fe_83_Ga_17_Er*_x_* alloys [29]. During electron back-scattering diffraction (EBSD) measurements, Kikuchi patterns were generated using an acceleration voltage of 20 kV, and recorded by means of a DigiView camera system (AMETEK, Leicester, UK) at a recording speed of the order of 0.1 s/pattern. Slightly longer time was required for multi-phase analysis. The working distance was 20 mm, and the step-size of the EBSD system was chosen to be 20 nm. More details regarding the measurement procedures may be found in References [30,31]. The results of the EBSD measurements are presented in the form of maps, the most important of which are the inverse pole figure (IPF) maps that indicate crystallographic orientation of individual foci.

Magnetic characterization was carried out using a vibrating sample magnetometer (Lake Shore, Model 7400, Westerville, OH, USA) in magnetic fields up to *H_app_* = ±1.2 T and in the temperature range (300 K ≤ *T* ≤ 1000 K). The magnetic transition temperature (*T_t_*) was determined as the inflection point in the derivative of magnetization (*M*) as a function of temperature (*T*) at an applied magnetic field of *μ*_0_*H* = 1 T. During magnetostriction measurements, the strain gauge was bonded longitudinally to the Galfenol samples in a quarter-bridge configuration to measure the magnetostrictive coefficient along the direction of growth of the samples. A Vishay Micro-measurement P3 strain Indicator was employed to measure the magnetostrictive strain, as the magnetic field was swept from 0 to 1 T at room temperature (~300 K).

## 3. Experimental Results & Discussion

### 3.1. Structural Attributes: Crystallographic and Microstructural Properties

**Figure 1:**
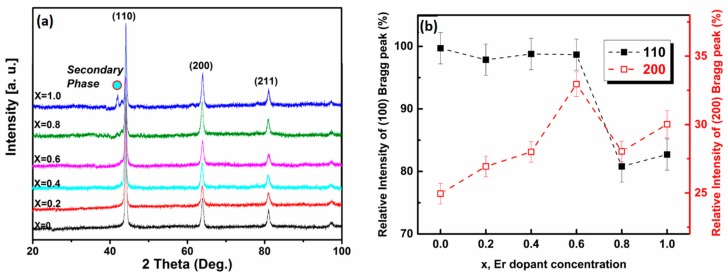
(**a**) X-ray diffraction pattern of directional solidified bulk alloys of Fe_83_Ga_17_Er*_x_* (0 < *x* < 1). An additional Bragg peak corresponding to a minor secondary phase is observed in samples where the Er dopant concentration (*x*) is higher than 0.6; (**b**) dependence of the relative intensity of the Bragg peaks corresponding to (110) and (200) planes on Er content (*x*).

X-ray diffraction data of the Fe_83_Ga_17_Er*_x_* alloys, obtained by scanning the sample plane perpendicular to the growth direction, as shown in Figure 1a, indicates the presence of a single phase having the bcc crystal structure for all samples of composition *x* < 0.6 (lattice parameter, *a* = 2.905 ± 0.005 Å). An additional Bragg peak corresponding to a minor secondary phase is observed at *2θ*~42°, as Er dopant concentration (*x*) is increased to *x* > 0.6. Overall, all the samples were found to be polycrystalline in character with a preferred orientation along the [110] growth direction—a feature attributed to the thermal gradient imposed by directional solidification. The dependence of relative X-ray intensity of the Bragg planes (110) and (200) with Er content is illustrated in Figure 1b. At all Er doping concentrations, the (110) plane retains dominance. Nonetheless, it is interesting to note that the (200) plane increases in intensity and displays the largest value for *x* = 0.6. It is hypothesized that Er favors occupation of the (200) planes at *x* < 0.6. These results are essential to further understanding the following measurements of magnetostriction as a function of Er content, as will be discussed in subsequent paragraphs.

**Figure 2:**
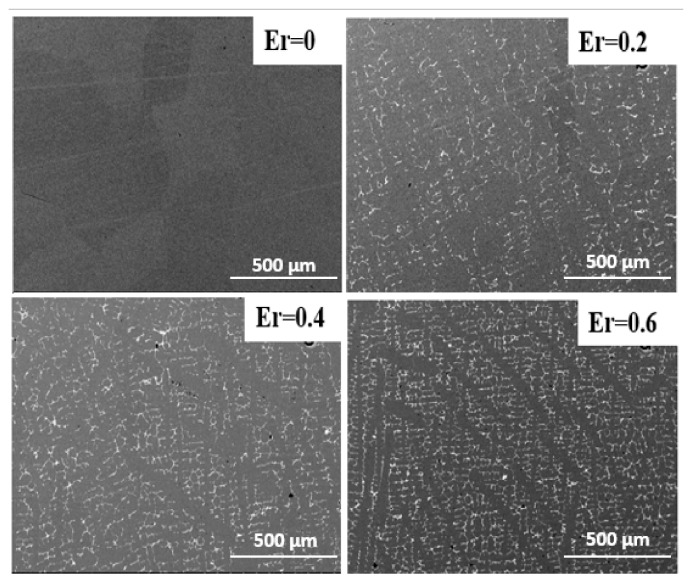
A microstructure of Er-doped (Fe_0.83_Ga_0.17_)_100−*x*_Er*_x_* (*x* = 0, 0.2, 0.4, 0.6).

The SEM images of select Fe_83_Ga_17_Er*_x_* alloys (0 < *x* < 1) are shown in Figure 2. The microstructure of the parent Fe_83_Ga_17_ alloy consists of a single solid solution with equiaxed grains of dimensions ~200 µm (Figure 2a). Conversely, the morphology of the Er-doped FeGa alloys demonstrates columnar grains mainly composed of a matrix phase (i.e., gray area) and precipitates of a secondary phase (i.e., white area) deposited along the grain boundary. The average size of the precipitates was approximately 1–3 μm, and it is observed that the fraction of precipitates increases with Er dopant concentration. Formation of the secondary phase in the Fe_83_Ga_17_Er*_x_* alloys is attributed to the realization that the atomic size of Er (175 pm) is significantly larger than that of Fe (140 pm) and Ga (135 pm). This difference in atomic dimensions leads to low solid solubility of Er in the FeGa matrix, and thus, Er exists mostly in the precipitates. This characteristic dual-phase microstructure has been observed previously in other rare-earth doped FeGa alloys [20,21,22,26,27], and it is considered to be advantageous for improving the magnetostrictive response and mechanical properties of this materials system. 

**Figure 3:**
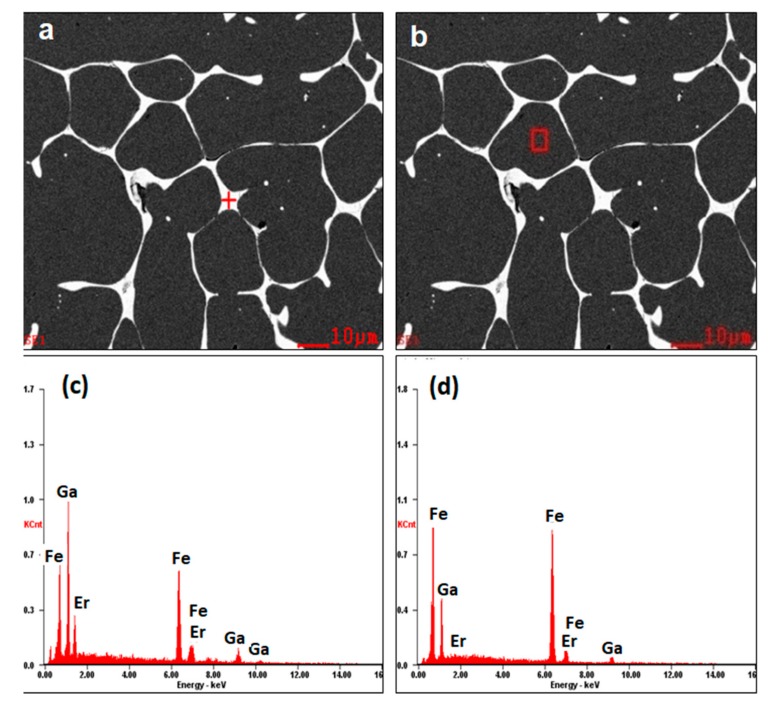
(**a**,**b**) SEM morphology of a sample of composition, Fe_83_Ga_17_Er_0.6_.; (**c**,**d**) EDXS profile of Fe, Ga and Er in the grain boundary region marked as “+” and in the intragranular region marked as “□”.

To determine the distribution of elements, the sample Fe_83_Ga_17_Er_0.6_ was analyzed by EDXS. Figure 3 shows SEM morphology and the determination of the constituent elements at spot “a” (grain boundary) and spot “b” (grain). It was found that the grains are composed primarily of Fe and Ga, while the grain boundary consists of Fe and Ga, as well as a relatively high percentage of Er. Stoichiometric determination using EDXS indicates that the Er atoms accumulate more in the grain boundary region and form an intermetallic secondary phase, possibly Ga_6_Er. Presence of the secondary phase in samples with relatively high Er concentrations is consistent with results obtained by X-ray diffraction analysis (see Figure 1).

**Figure 4:**
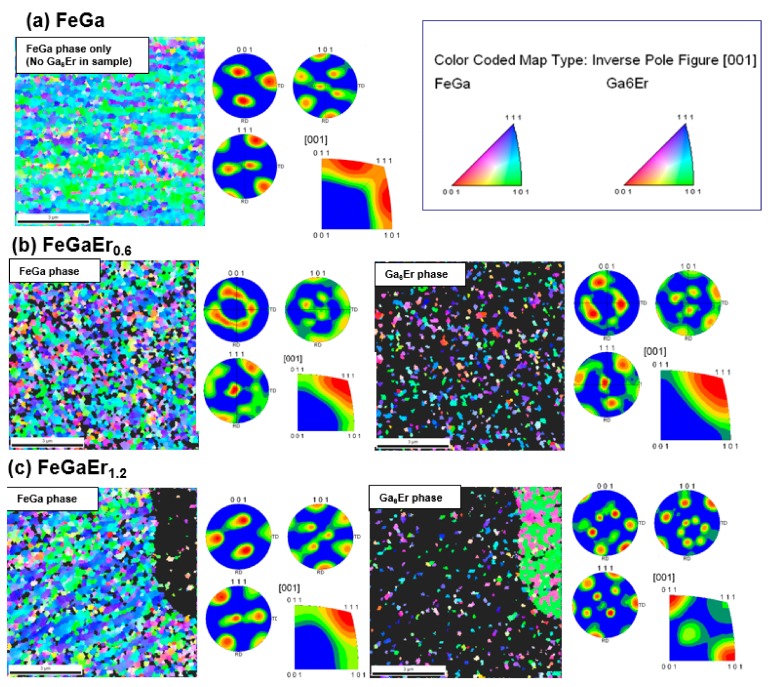
Inverse pole figure maps and corresponding pole figures for FeGa and the Ga_6_Er phases in samples of the following compositions: (**a**) Fe_83_Ga_17_ (parent compound); (**b**) Fe_83_Ga_17_Er_0.6_ and (**c**) Fe_83_Ga_17_Er_1.2_. The inverse pole figures shown here give the crystallographic orientation of the grains, according to the stereographic triangles for each phase.

Information regarding the texture of the Fe_83_Ga_17_Er*_x_* alloys (0 < *x* < 1.2) was obtained using EBSD analysis. Kikuchi patterns obtained using this technique were indexed, using material files pertaining to FeGa (based on bcc α-Fe structure; Pearson symbol: cI2) and Ga_6_Er (based on tetragonal crystal structure; Pearson Symbol: *tP*14). Here, it should be noted that we could observe the Ga_6_Er phase from the sample with *x* = 0.2. This demonstrates the importance of a highly spatially resolved measurement. Figure 4 shows the IPF maps along the [001] direction (i.e., perpendicular to the sample surface) and corresponding pole figures for the following compositions: Fe_83_Ga_17_ (i.e., the parent compound), Fe_83_Ga_17_Er_0.6_ and Fe_83_Ga_17_Er_1.2_. The IPFs shown here provide the crystallographic orientation of the grains, according to the stereographic triangles for each phase. In confirmation with results obtained by X-ray diffraction and SEM-EDS, no Ga_6_Er phase was observed in the parent Fe_83_Ga_17_ compound (Figure 4a). In Fe_83_Ga_17_Er_0.6_, the Ga_6_Er precipitates were present, as indicated as small spots scattered over the scan area (Figure 4b). Conversely, significant aggregation of Ga_6_Er was also found in Fe_83_Ga_17_Er_1.2_ (Figure 4c). The IPF maps and the pole figures indicate that while the Fe_83_Ga_17_ parent compound demonstrates [001] texture, Fe_83_Ga_17_Er_0.6_ and Fe_83_Ga_17_Er_1.2_ samples do not exhibit preferred grain orientations. It is important to realize that grain size of both the FeGa and the Ga_6_Er phases in the Fe_83_Ga_17_Er*_x_* samples increased as a function of the Er concentration. As the dopant concentration was increased, Ga_6_Er formed a secondary phase in the grain boundary region (Figure 4b,c), and the orientation of the grains is found to be in [001] and [101] directions. When the Ga_6_Er is located within the matrix, the grains have the same orientation as the matrix—a feature attributed to the coherent relationship between the two phases. As the grain size of the FeGa and the Ga_6_Er phases increases, the crystallographic orientation between these phases became significantly different (see pole figures in Figure 4c), and thus, it is inferred that the secondary phase precipitates were randomly oriented in the matrix phase in the large grained Fe_83_Ga_17_Er*_x_* alloys.

**Figure 5:**
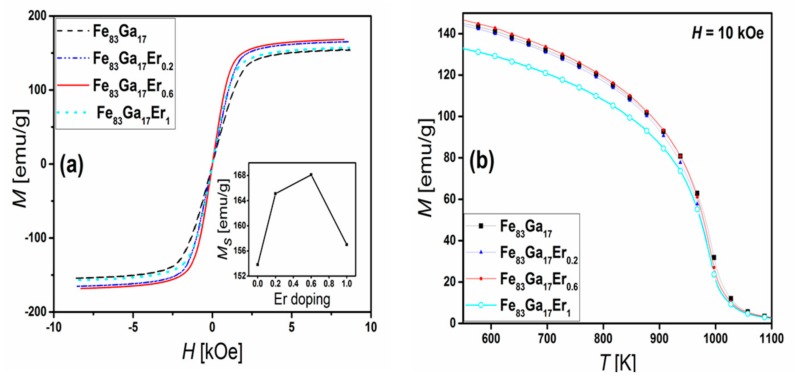
(**a**) Magnetization hysteresis loops of Fe_83_Ga_17_Er*_x_* (*x* = 0, 0.2, 0.6, 1). The inset shows the saturation moment as a function of the Er doping amount; (**b**) Magnetization (emu/g) of (Fe_0.83_Ga_0.17_)_100−*x*_Er*_x_* at *H* = 10 k Oe as a function of temperature for different amounts of Er doping.

Addition of Er has noteworthy effects on the magnetic properties of [110]-textured polycrystalline FeGa alloys. The magnetization behavior of Fe_83_Ga_17_Er*_x_* alloys (0 < *x* < 1.5) as a function of a magnetic field at room temperature (*T* = 300 K), shown in Figure 5a, indicates that doping with Er increases the saturation magnetization (*M_s_*) by approximately 10%, as compared to the Fe_83_Ga_17_ parent alloy. As shown in the inset of Figure 5a, *M_s_* initially increases from 153.8 to 168.2 emu/g, as the Er content increased from 0 to 0.6. Further increase in Er doping decreases *M_s_* slightly—a feature attributed to the significant presence of the secondary Ga_6_Er phase. The temperature-dependent magnetization behavior of the Fe_83_Ga_17_Er*_x_* samples at an applied magnetic field of *H* = 1000 Oe is shown in Figure 5b. Consistent with previous reports, the Curie temperature of Fe_83_Ga_17_ was found to be approximately 990 K [8]. Overall, the *T_c_* of the Fe_83_Ga_17_Er*_x_* samples was found to be independent of the Er concentration.

### 3.2. Enhanced Functional Response: Magnetostriction Measurements at Room Temperature

**Figure 6:**
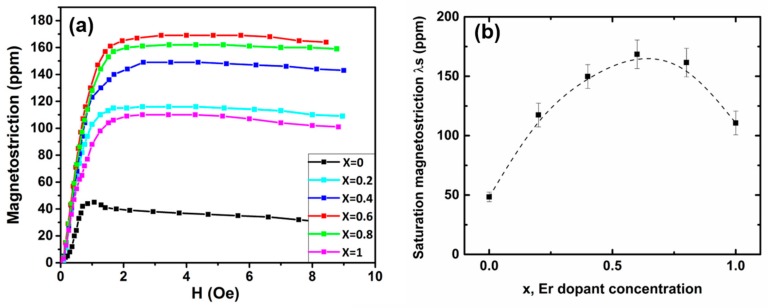
(**a**) Magnetostriction of the (Fe_0.83_Ga_0.17_)_100−*x*_Er*_x_* (0 < *x* < 1) alloys as a function of the applied magnetic field. An enhanced saturation magnetostriction value (*λ_s_*) of more than 250% is measured relative to the parent compound; and (**b**) change in *λ_s_* as a function of the Er dopant concentration (*x*).

The magnetostriction strain (*λ*) along the direction of growth of the Fe_83_Ga_17_Er*_x_* alloys is plotted as a function of an applied magnetic field in Figure 6a. In all samples, *λ* increases with applied magnetic field until a saturation magnetostriction value (*λ_s_*) is obtained. In agreement with previous studies on directionally solidified [110]-textured FeGa alloys [21], the *λ_s_* of Fe_83_Ga_17_ was found to be ~45 ppm. Change in *λ_s_* with Er content (*x*) for the Fe_83_Ga_17_Er*_x_* alloys is shown in Figure 6b. Overall, *λ* increased with Er doping till the maximum of 170 ppm was achieved at *x* = 0.6. These results represent a record enhancement of more than ~275% in *λ_s_* of Fe_83_Ga_17_Er*_x_* alloys with the introduction of small amounts of Er. For operation in actuators and sensors in low loss magnetoelectric and multiferroic devices, such as those described in References [32,33,34,35,36], magnetostrictive materials must be operated under mechanical and magnetic bias conditions to achieve the maximum strain per unit magnetic field. Thus, permeability (*µ*) as realized from the derivative of magnetization/magnetostriction with respect to applied magnetic field ($dM/dH_{app}$ or $d\lambda /dH_{app\;}$) is a desired figure of merit in magnetostrictive materials. To this end, it is critical to observe from Figure 6a that the low field derivative of magnetostriction with respect to the applied magnetic field ($d\lambda /dH_{app}$ for *H_app_* up to 1000 Oe) increases by ~230% with Er doping ($d\lambda_{s}/dH_{app,\mathrm{FeGa}}=$ 0.045 ppm/Oe; $d\lambda_{s}/dH_{app,\mathrm{FeGaEr}}=$ 0.15 ppm/Oe).

For FeGa single crystals, a maximum in magnetostriction is reported along the easy magnetic axis, i.e., along the <100> crystallographic direction. Assuming the approximation of only dipole–dipole interactions within the material, the magnetostriction values for a [110]-textured polycrystalline materials may be calculated using the expression [8]:

(1) $$\lambda_{110}=\frac{1}{4}\lambda_{100}+\frac{3}{4}\lambda_{111}$$

where *λ*_100_ and *λ*_111_ are the saturation magnetostriction when the crystal is magnetized and the strain is measured along the <100> and <111> directions, respectively. In the absence of compressive pre-stress, the calculated values of (3/2)*λ*_100_ and (3/2)*λ*_111_ for Fe_83_Ga_17_ are ~311 ppm and −20 ppm, respectively [37]. Note, that the factor 3/2 arises from the definition of magnetostriction as a deformation from a demagnetized state [37]. The theoretical (3/2)*λ*_110_ value for a polycrystalline Fe_83_Ga_17_ sample was in approximate agreement with our experimentally determined magnetostriction value of *λ*_110_ = 45 ± 5 ppm. It is critical to note that the use of Equation (1) for the Fe_83_Ga_17_Er*_x_* alloys was based on the broad postulation that the texturing degree of the doped samples is similar to that of the pure Fe_83_Ga_17_ sample.

The (3/2)*λ*_110_ results of [110]-textured rare-earth doped FeGa alloys is summarized in Table 1 with relevant references. It is important to note that with an exception of one report concerning Fe_83_Ga_17_Ce_0.8_ [22], the Fe_83_Ga_17_Er*_0.6_* sample demonstrates higher magnetostriction than any other [110]-textured directionally-solidified FeGa alloy synthesized to date.

At present, the enhanced magnetostrictive response of the doped Fe_83_Ga_17_Er*_x_* alloys is attributed to a combination of electronic and microstructural effects. Previous studies on binary FeGa alloys, conducted by Clark et al., suggest that the large magnetostriction in this intermetallic alloy originates from local magnetocrystalline anisotropy, induced by local short-range interactions between the Ga atoms along specific crystallographic directions in the disordered bcc α-Fe structure [37]. It is likely that a large number of *4f* valence electrons and aspherical charge cloud distributions observed in Er atoms lead to enhanced magnetic anisotropy due to crystalline electric field effects. Considering that Er possesses a larger atomic radius (178 pm) relative to Fe (127 pm) and Ga (140 pm), it is possible that the strain due to local lattice distortions influence the magnetic properties of the Fe_83_Ga_17_Er_x_. In related compounds, namely Tb- and Ce-doped directionally solidified FeGa systems, an increase in magnetostriction has been linked to improved grain orientation and morphology [23,24]. Based on experimental and computational studies conducted by He et al., it is surmised that the giant magnetostriction in rare-earth doped FeGa alloys may be ascribed to the presence of nano-heterogeneties in the samples [28]. The dopants tend to enter the nano-heterogeneities, creating a larger tetragonal distortion of the matrix, as well as increased magnetocrystalline anisotropy. A mesoscopic model developed using phase field simulations shows that the bulk tetragonal distortion arises mainly from those nano-heterogeneities with fixed Ga–Ga pairs parallel to the applied magnetic field [28].

It is worth discussing the experimental results obtained in this study in the context of the phenomenological model reported by He et al. to predict magnetostrictive trends in FeGa alloys doped with rare-earth elements [24]. According to this model, the elements (i.e., Ce, Pr and Tb) have the greatest impact on magnetostriction of FeGa among all the rare-earth elements, due to the negative rare-earth quadrupole moment and local lattice tetragonal distortion of the matrix, which is triggered by the heterogeneous nanostructure with local tetragonal distortion [24]. If this were indeed true, the magnetostrictive behavior of the directionally solidified [110]-textured Fe_83_Ga_17_Tb*_x_* compounds investigated by Fitchorov et al. [34] and Jiang et al. [20] would be greater than that of the Fe_83_Ga_17_Er*_x_* samples examined in the current study. However, our experimental results suggested otherwise. Insight into a potential explanation for this contradiction may be obtained by comparing structure-magnetic property correlations between Fe_83_Ga_17_Er*_x_* and Fe_83_Ga_17_Tb*_x_* alloys. In both materials systems, only trace amounts of rare-earth dopants were required for optimal magnetostrictive performance in the Fe_83_Ga_17_Er*_x_* and Fe_83_Ga_17_Tb*_x_* systems. Excessive doping destroys magnetostriction, due to limited solid solubility of the rare-earth element in the FeGa lattice and subsequent formation of the intergranular secondary phases. The maximum magnetostriction in the [110]-textured directionally solidified Fe_83_Ga_17_Er*_x_* and related Fe_83_Ga_17_Tb*_x_* system was observed at *x* = 0.6 and 0.2 respectively, and thus, it is speculated that the solid solubility of Er in FeGa may be slightly more than that of Tb. It is further hypothesized that enhancement of magnetostriction may be achieved either by the application of a compressive pre-stress or by increasing the solid solubility of Er in the bcc FeGa matrix through quenching during the cooling phase of the sample fabrication technique. Indeed, previous studies in the FeGa literature demonstrate that the saturation magnetostriction of Fe_83_Ga_17_ alloys can be remarkably increased by the melt spinning method [22,38]. It is, however, critical to note that it is difficult to experimentally measure the magnetostriction of melt-spun ribbons, as the grains in the sample usually grow perpendicular to the direction of sample growth [22]. Moreover, due to large demagnetizing effects, shape anisotropy in FeGa ribbon samples typically leads to high magnetic saturation fields [38]. From the perspective of user inspired research, the directional solidification technique is more amenable for commercial production of rare-earth doped FeGa alloys. 

## 4. Conclusions

In summary, in this work we present the effects of Er additives upon the microstructure, magnetic and microstructural properties of Fe_83_Ga_17_Er*_x_* alloys prepared by vacuum arc-melting and directional solidification methods. Data obtained in this experimental study indicate a room temperature magnetostriction value of 170 ppm in a [110]-textured polycrystalline sample of nominal composition Fe_83_Ga_17_Er_0.6_—a value that is ~275% higher than that of the corresponding parent Fe_83_Ga_17_ compound. Overall, addition of small amounts of Er into the FeGa lattice results in an increase in saturation magnetization and magnetostriction and a reduction in the saturation field. These characteristics are beneficial for practical applications, such as actuators in multiferroic magnetic field generators that use a converse magnetoelectric effect or high-sensitivity magnetic field sensors that operate based on the direct magnetoelectric effect without the need for a bias DC field. The enhanced magnetostrictive response of the Fe_83_Ga_17_Er*_x_* alloys is ascribed to an amalgamation of electronic and microstructural factors, namely: (i) strong local magnetocrystalline anisotropy due to the large spin-orbit coupling and the highly anisotropic localized nature of the *4f* electronic charge distribution of the Er atom, (ii) improved grain orientation and morphology due to deposition of Er in the intergranular region and (iii) local strain effect that may arise due to incorporation of Er into the FeGa lattice. Excessive Er doping destroys the improved magnetostriction in Fe_83_Ga_17_Er*_x_* alloys, due to formation of an undesirable secondary phase, which is identified as the intermetallic compound, Ga_6_Er. Overall, these results highlight the potential for modifying the functional response of FeGa alloys by addition of tiny amounts of the rare-earth element, Er. To further understand the origin of the superior functional response of rare-earth doped FeGa systems, future work involving computational modeling of the magnetostrictive behavior of these compounds is desired.

## Figures and Tables

**Table 1:** Magnetostriction coefficients of [110]-textured rare-earth doped FeGa alloys synthesized via the directional solidification technique.

Alloy	Fabrication Technique	*λ*_110_ (ppm)	Condition	References
Fe_83_Ga_17_	Directional solidification	45	Bulk; Pre-stressed	Current work
Fe_83_Ga_17_	Directional solidification	68	Bulk; Pre-stressed	L. Jiang et al. [20]
Fe_81_Ga_19_Tb_0.3_	Directional solidification	85	Bulk; Pre-stressed	T.I. Fitchorov et al. [34]
Fe_83_Ga_17_Y_0.64_	Directional solidification	133	Bulk; Compressed under 15 MPa	L. Jiheng et al. [23]
Fe_83_Ga_17_Tb_0.2_	Directional solidification	160	Bulk; Pre-stressed	L. Jiang et al. [20]
Fe_83_Ga_17_Er_0.6_	Directional solidification	170	Bulk; Pre-stressed	Current work
Fe_83_Ga_17_Ce_0.8_	Directional solidification	200	Bulk; Pre-stressed; Sample not at saturation	Z. Yao et al. [22]
